# Dead Reckoning in the Desert Ant: A Defence of Connectionist Models

**DOI:** 10.1007/s13164-014-0180-9

**Published:** 2014-02-22

**Authors:** Christopher Mole

**Affiliations:** Department of Philosophy, University of British Columbia, Vancouver, V6T 1Z1 Canada

## Abstract

Dead reckoning is a feature of the navigation behaviour shown by several creatures, including the desert ant. Recent work by C. Randy Gallistel shows that some connectionist models of dead reckoning face important challenges. These challenges are thought to arise from essential features of the connectionist approach, and have therefore been taken to show that connectionist models are unable to explain even the most primitive of psychological phenomena. I show that Gallistel’s challenges are successfully met by one recent connectionist model, proposed by Ulysses Bernardet, Sergi Bermúdez i Badia, and Paul F.M.J. Verschure. The success of this model suggests that there are ways to implement dead reckoning with neural circuits that fall within the bounds of what many people regard as neurobiologically plausible, and so that the wholesale dismissal of the connectionist modelling project remains premature.

## Introduction

In a series of works, co-authored with several collaborators, C. Randy Gallistel has argued that the explanation of certain elementary psychological phenomena requires the postulation of an addressable read/write memory in the brain. He has argued that such a memory could not plausibly be implemented by a mechanism of the sort that dominates the theories of information storage given by current neuroscience, and so that something radically new will need to be discovered in neuroscience, before these phenomena can be explained. The most extended presentation of this argument is in Gallistel and King 2009.

This line of thought is bad news for any research in computational neuroscience that adopts a broadly connectionist approach. The elements from which the connectionist attempts to build her models of psychological phenomena are elements that behave in ways corresponding to what our current neuroscientific theories do successfully explain. If Gallistel is right, and the theoretical resources of current neuroscience are explanatorily inadequate, then connectionism’s deployment of those resources in the course of its psychological model building must be explanatorily limited. Gallistel’s argument has therefore been thought to be a fatal blow to connectionism’s long-ailing research programme.

Although I am persuaded that something along the lines of Gallistel’s main claim must indeed be correct when it is applied to cognitive phenomena with any degree of sophistication, I am not convinced that the news for connectionism is altogether as bad as Gallistel’s writings might lead one to suppose. A central part of Gallistel’s argument is his attempt to show that the connectionist’s explanatory resources are unable to account even for such primitive psychological accomplishments as the dead-reckoning behaviour of the desert ant. The point that I argue for here is restricted to that particular case. I show that recent models of dead reckoning illustrate some explanatory tactics that the connectionist has available, and that enable her to avoid the sorts of problem that Gallistel raises. I argue that these models show connectionism can do a better job of explaining the desert ant’s dead-reckoning behaviour than Gallistel allows. I do this with the intention of advertising the strength of these tactics, but without taking a stance on how widely applicable they are.

## Dead Reckoning

When foraging, a desert ant will take a more or less randomly winding course until it happens upon some food. It will then navigate directly back to its nest, even in the absence of landmarks indicating where that nest is. In order to do this the ant must be integrating the several segments of its winding path, so that it can keep track of the direction home. It must therefore be capable of dead reckoning (Wehner 2003; Gallistel and King 2009, pp. 196–197). The question of how the ant performs dead reckoning is an open one, although several models that attempt to account for this ability have been proposed (e.g., Droulez and Berthoz 1991; Vickerstaff and Di Paolo 2005; Haferlach, et al. 2007).

Chapter fourteen of Gallistel and King’s 2009 book, *Memory and The Computational Brain*, is devoted to showing that connectionist approaches to the explanation of dead reckoning are unsatisfactory, and that they are unsatisfactory for reasons that are essential to the connectionist approach, so that something radically different must be called for. These arguments are directed primarily against the model of dead reckoning presented in a 1997 article by Alexei Samsonovich and Bruce L. McNaughton. My tactic here is to show that these arguments do not apply to more recent connectionist models of dead reckoning. For concreteness, and because it is unusually straightforward in its operation, I shall focus on the model of dead reckoning presented in a 2008 article by Ulysses Bernardet, Sergi Bermúdez i Badia, and Paul Verschure. Similar points could be made with reference to other models that have been proposed (such as Cruse and Wehner 2011).

Before we turn to the details of the Bernardet et al. model, let me take a moment to be clear about what I am and am not objecting to. Gallistel and King’s main business is to show that the postulation of a read-write memory is indispensible in the explanation of even quite simple psychological phenomena. My objection is not to that, but to the idea that postulating some such memory will require mechanisms of a sort that neuroscientists have not yet identified in the brain, and that are therefore anathema to the project of connectionist modelling. I shall be suggesting that the kind of memory required for dead reckoning is not especially complex; that it can be modelled in a neural network that suffers from none of the *ad hoc* implausibilities that Gallistel and King have exposed; and that it can be modelled without postulating any as-yet-unknown neural mechanisms for storing information in a format where trigonometric functions (or even such functions as multiplication) can be performed upon it.

In what follows I briefly explain the trigonometry-free way in which the Bernardet et al. model of dead reckoning works. I then set out Gallistel and King’s objections to the connectionist approach, which are mostly targeted at the Samsonovich and McNaughton model. In the course of examining these objections, I show why it is that they do not apply to the model of Bernardet et al. Finally, I attempt to explain how the Bernardet model achieves what it does, and to draw some lessons concerning the philosophical treatment of the connectionist project.

Although I shall show that the Bernardet et al. model does not involve the implausibilities that Gallistel and King have identified, I do not attempt to address all possible objections to that model. It is open to Gallistel and King to complain that the mechanisms employed in this model are still implausible ones, although for reasons other than those that motivated their previous objections. There is an ongoing conversation that might be had about that model’s ultimate plausibility. My assertion is that Gallistel and King’s existing arguments do not reveal that conversation to be futile, and that ongoing neuroscientific and philosophical engagement with it should be regarded as a part of normal science. The difficulties of accounting for dead reckoning should not be regarded as Kuhnian ‘anomalies’, of the sort that might indicate the need for a whole new neuroscientific paradigm. There is work remaining to be done, if we are to show whether and how the Bernardet model could be plausibly implemented, but there is not yet any reason to think that normal scientific resources will be unable to do that work. The case for a neuroscientific paradigm shift is better made by reference to psychological phenomena that are more complex than desert ant navigation.

## The Bernardet Model

**Fig. 1:**
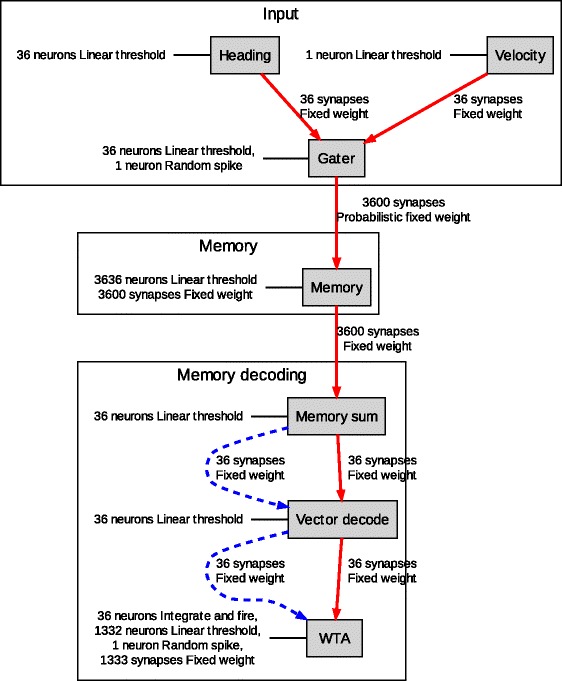
Reprinted with kind permission from Springer Science+Business Media (Bernardet et al. 2008; Fig. 4)

The basic architecture of Bernardet et al’s model of dead reckoning is represented in Fig. 1.

As is standard with connectionist models, Bernardet et al’s system treats time as passing in a series of discrete steps. At each time step, the model takes as input a representation of the direction in which the creature is heading at that moment, together with a representation of the speed at which the creature is travelling. The heading is represented to the nearest 10°, by activating the appropriate neuron in a group of thirty six neurons (each of which can be thought of as corresponding to one of the compass bearings on a thirty six point compass). The group containing these thirty six neurons is shown in the top left of Fig. 1. The speed of the creature’s travel at each time is represented, at that time, by the activity level of a single cell (shown in the top right of the Fig. 1).

In order to model the ant’s capacity for dead reckoning, each of these several steps of heading and speed information will need to be integrated, to give a representation of the overall heading for the total journey. This heading can then be inverted to give the direction back to the nest.

The model’s first step is to combine the heading and speed information in a single subsystem (labelled ‘Gater’ in Fig. 1). This subsystem again has one neuron for each of the thirty six compass points, as did the Heading subsystem, but in this Gater subsystem the neuron corresponding to the direction of the creature’s current travel now fires at a rate proportional to the representation of the creature’s current velocity. The firing of any one cell in this Gater subsystem goes on to activate cells in one of thirty-six corresponding columns in a separate Memory system.

Each column in this Memory system contains one hundred neurons. All one hundred neurons in each column of the system receive input from one of the neurons in the Gater subsystem. The point that is crucial to the way in which this model operates is that these connections between cells in the Gater subsystem and cells in the Memory subsystem are stochastic: The firing of a cell in the Gater subsystem has a 5 % chance of activating any given cell in the corresponding column of the Memory system. The incorporation of stochastic connections at this point in the model is a deviation from the old-fashioned connectionist models, with which some philosophers may be more familiar. The connections in more old-fashioned connectionist models transmitted information in a weighted but surefire fashion. The more recent use of stochastic connections is clearly a step in the direction of biological plausibility. It is one of the tactics that adds to the computational efficiency of the model under consideration.

Another deviation from traditional connectionism – and again a crucial one – is that, once activated, the cells in each column of the memory system will remain active. We shall return to the biological plausibility of this feature in the discussion that follows, but it should at least be clear that some persistent firing of neurons is not entirely remote from the sort of thing that our current neuroscience is able to explain. Guy Major and David Tank, in a 2004 review of persistent neural firing’s prevalence and possible mechanisms, write that:

Persistent neural activity […] is found in a diverse set of brain regions and organisms and several in vitro systems, suggesting that it can be considered a universal form of circuit dynamics that can be used as a mechanism for short-term storage and accumulation of sensory or motor information. (p. 675)

Bernardet et al. use persistent firing in just such a short term storage and accumulation role. Because cells in the Memory system remain active once they have been activated, and because there are several such cells in each column of that system, each one of which is activated with a low probability by the firing of a corresponding cell in the Gater subsystem, the number of cells firing in any given column of the Memory system will be proportional to the total distance that has been travelled in the corresponding compass direction.

The pattern of sustained firing across all the columns of the Memory system therefore encodes information about the total distances travelled in each of the thirty-six different directions. This pattern-encoded information can then be used, once the outward journey is complete, to generate a representation of the total integrated path that has been travelled (and so it can be used to accomplish dead reckoning). In the Bernardet et al. model this calculation of the total integrated path is achieved via a three-stage process (although one could just as successfully achieve it with fewer stages, if one were willing to employ more synapses).

The first of the three stages maps each one-hundred cell column of the Memory system onto a single cell in the ‘Memory Sum’ subsystem, so that this ‘Memory Sum’ cell now has an activity that is proportional to the number of active cells in the corresponding column of the Memory system. At stage two of the memory decoding process every cell in the Memory Sum subsystem is then connected to every cell in the ‘Vector Decode’ subsystem (in the middle of the bottom section of the diagram given in Fig. 1). Each cell in this Vector Decode subsystem therefore receives input from all thirty-six cells in the Memory Sum subsystem. These inputs are in some case inhibitory and in others excitatory. More precisely, the activity of each Vector Decode cell is strongly excited by activity from one of the Memory Sum cells; is somewhat less strongly excited by activity from each of the adjacent Memory Sum cells; is even less strongly excited by the Memory Sum cells that are yet further out; and is actively inhibited by the cells that are furthest away. The rate at which excitation drops off and inhibition comes in is determined by a simple Gaussian function.

The third and final step of the memory decoding process is a winner-takes-all operation (labelled ‘WTA’, in the Fig. 1). This determines which of the cells in the Vector Decode subsystem is most active. That most-active cell gives the direction of the total integrated path, and so it can be used to give the direction back to the point where the journey began.

When Bernardet et al. tested this model on the information about velocities and headings that they had taken from the actual paths of ants, observed in an experimental chamber, (and also when they tested it on more systematic and artificial inputs) the network succeeded in identifying the direction to the start of the total integrated path with a margin of error in the region of 20°. That is comparable to the margin of error observed in actual ants (Müller and Wehner 1988, figure 4). To that extent this network provides an accurate model of dead reckoning in the ant. Bernardet et al. note that the level of activation in the Memory system also encodes information about the total distance travelled, which might perhaps be useful in modelling the ant’s success in knowing how *far away* the nest is. They do not attempt to develop that aspect of the model.

## The Gallistel and King Arguments

Gallistel and King’s *Memory and The Computational Brain* was published in 2009, and is based partly on lectures that were given by Gallistel in 2003. It does not address Bernardet et al’s 2008 paper. Gallistel and King’s complaints against connectionist approaches to dead reckoning are instead directed at a rather different model, developed by Samsonovich and McNaughton 1997. Gallistel and King are clear that they are not intending to attack only Samsonovich and McNaughton. They are instead taking the Samsonovich and McNaughton model to be “representative of a large class of models” (p. 265), writing that:

This model relies on the only widely conjectured mechanism for performing the essential memory function, reverberatory loops. We review this model in detail because it illustrates so dramatically the points we made earlier about the price that is paid when one dispenses with a read/write memory. To our mind, what this model proves is that the price is too high. (p. xv)

We shall consider Gallistel and King’s several complaints against this model in turn.

Gallistel and King’s first complaint against Samsonovich and McNaughton’s model is based on that model’s size and complexity. The model uses large arrays of neurons to represent the creature’s previous and present locations. In each of these arrays each neuron has excitatory connections with all of its neighbours, and inhibitory connections with all of its non-neighbours. This requires a very large number of connections within each array, leading Gallistel and King to complain that:

There is a question how much credence one should give to the reported success of simulations like this, because of the enormous complexity of the models and the resulting complexity of the computer programmes that simulate them. (Recall that there are more than two billion connections within the P array alone [that is, within the ‘place representing array’], which is but one of seven interconnected large arrays.) (p. 254).

Since the ants that successfully perform dead reckoning have nervous systems with a number of neurons on the order of hundreds of thousands, Samsonovich and McNaughton’s need for billions of connections in their model is indeed problematic, just as Gallistel and King say. But sheer number of connections cannot be the basis for any objection against the Bernardet et al. model that we reviewed above. That model employs a total of 5,151 neurons, with a total 5,187 synapses (Bernardet et al. op cit. p. 169). For a model of the ant’s navigation system, this is a plausible order of magnitude.

Notice also that increasing the performance of the Bernardet model – by using more than thirty six compass points to encode directions, or by increasing the number of cells in each of the memory columns, so that longer paths could be integrated – would lead to only a small, polynomial increase in the number of neurons and connections required. The model is both small and scalable. Unlike the Samsonovich and McNaughton model it does not require implausibly complex computational resources.

Gallistel and King’s second complaint is that, in order to avoid the problem of having to deal with edges, the Samsonovich and McNaughton model represents the space through which the creature is navigating as if it were a space that has no edges. They achieve this by the patently *ad hoc* device of supposing that the creature whose dead reckoning behaviour is being modelled lives on the surface of a torus. Gallistel and King question whether there is any plausible implementation of the model that avoids the need to employ such *ad hoc* fixes. In the Bernardet *et al*. model, however, no such *ad hoc* fixes are required, since no problem of having to deal with edges arises. That model represents the creature’s headings and velocity, but, as we have seen, it contains no explicit representations of that creature’s location within the space that it is navigating. The Bernardet *et al*. model therefore has no need for any suppositions, *ad hoc* or otherwise, about the topology of that space.

In each of these cases the model benefits from a tactic that has often been advocated by proponents of ‘extended cognition’ (e.g., Clark 1999), and of ‘the new AI’ (e.g., Brooks 1991): It dispenses with the need to form a representation of its environment, and of its location within it. Instead the model stores the minimal information required to coordinate its action.

Gallistel and King’s third complaint against Samsonovich and McNaughton is similarly inapplicable to the Bernardet *et al*. model. We have said that the arrays in the Samsonovich and McNaughton model contain a large number of place cells. The creature’s location at any one time is encoded by making some localized cluster of these cells active. In order to ensure that the cells in these arrays are always activated in such localized clusters, the Samsonovich and McNaughton model employs a mechanism in which activation in one part of the array inhibits activity elsewhere. In implementing this mechanism one faces difficulties of timing, associated with arranging for the simultaneous inhabitation of all non-local parts in a large array. Gallistel and King set out some of these difficulties as follows:

The computation [of this inhibitory activity] would have to track each impulse as it propagated from each neuron to all the neurons inhibited by that neuron. Because these neurons lie at different distances from the source neuron, the spikes will arrive at them at different times. And then one must compute the time course of the inhibitory postsynaptic action of each arriving impulse […] The amount of computation that must be done rapidly gets out of hand. (p. 256)

In the actual implementation of their model Samsonovich and McNaughton dodge these difficulties by adding a hard constraint on the amount of activity in each array. Instead of implementing an inhibitory mechanism to bring it about that activity in their place arrays is always localized, they simply restrict the model in such a way that each array cannot have more than some small amount of activity in it. “This” say Gallistel and King, “is an understandable shortcut.” But they are suspicious about whether there is any biologically plausible mechanism by which such shortcuts can be avoided. So long as some such shortcut is being employed: “There is a god outside the machine, mediating communication between its elements in a manner that frees communication from ordinary constraints of time and space and the messiness that those constraints can produce in a complex signalling system” (p. 256).

The Bernardet *et al*. model does not face the difficulty that motivates the introduction of this shortcut, and so the problem that such shortcuts create cannot be used as the basis for an objection against it. As the above quotation from Gallistel and King makes clear, the difficulty that motivates Samsonovich and McNaughton’s shortcut arises when arrangements have to be made for large numbers of signals to arrive at one place at one time, despite the fact that those signals are arriving from different sources, situated at different distances from their destination. We can see relatively easily that the architecture of the Bernardet et al. model does not require any such coordination.

There are only two places in that model at which information from multiple sources has to arrive at a single destination. The first of these is in the input system, where the Gater subsystem has to combine information from the Heading subsystem and from the Velocity subsystem. These two pieces of information do need to be combined in a time sensitive manner, so that the heading information is combined with information about the speed at which the ant was travelling *at the time when in was travelling in the relevant direction*. But this does not involve the difficulties of combining large numbers of signals from disparate sources. There are only two signals needing to be combined here: in the Velocity subsystem speed is represented in the activity of a single neuron (it would more properly be called a Speed subsystem), and in the Heading subsystem there is only one neuron that is active at any one time. The Gater subsystem need only coordinate these two signals. This is not a problematically complex task.

The only other place in the Bernardet et al. model at which multiple signals need to be coordinated is in the Vector Decode subsystem, in which each cell has to weigh the several inhibitory or excitatory signals that it receives from the thirty six neurons in the Memory Sum subsystem. Here there are several signals all needing to be responded to within a single time step, but again there is no difficulty in coordinating their arrival, since in this case these several signals are all coming from a single source. Combining inhibitory and excitatory signals from across the thirty-six cells of the Memory Sum subsystem therefore has none of the computational complexity involved in combining inhibitory and excitatory signals from across the 45,000 neurons in Samsonovich and McNaughton’s P array.

Gallistel and King’s fourth complaint is that Samsonovich and McNaughton do not tell us whether their network can cope with noise. They suggest that this is a bad oversight, since some network architectures are known to perform very badly when noise is introduced (p. 256). That latter suggestion is perhaps a little unfair. Although it is true that some networks perform badly when noise is introduced, it would be a mistake to suppose that noise is always a serious threat to the performance of connectionist networks, or that such networks should always be presumed to be fragile to noise until proven otherwise. There are existing connectionist models of dead reckoning that *have* been tested in noisy conditions, and they are found to perform well in them (e.g. Haferlach, et al. 2007). Although Bernardet et al. fail to report any trials in which their system is shown to cope with noise, and although this failure might be thought of as a shortcoming of their presentation, there are reasons to be optimistic that their model could indeed cope with noise successfully.

The first of these reasons is that the Memory system accumulates a sampling of all the heading and speed information during the ant’s entire walk, and then integrates all of this information by weighing it together, when feeding the activation from the cells of the Memory Sum subsystem down into the Vector Decode subsystem. Since any one bit of heading and speed information is thereby weighed against all of the others, there is reason to think that introducing noise anywhere in the network’s input system would not lead to a disproportionate loss of performance. The introduction of noise would, of course, impair the system’s performance to some degree, but there is no reason to think that introducing it in the input system would impair that performance disproportionately, since any noise introduced there will always come to be weighed against all of the signal.

Nor does it seem likely that the introduction of noise to the Memory system would be disproportionately disruptive, since this system is already a stochastic one, in which each individual cell picks up information from the Gater subsystem in a random fashion, with a low probability. One could introduce noise here by tweaking some of these probabilities, or by arbitrarily turning on or off some scattering of the memory cells. Doing so could not cause the system to go into a state such as it would never encounter when running without noise, since, even when noise is absent, these cells are already turning on somewhat randomly. That provides some reason to think that introducing noise in this system would not break down the system’s performance disproportionately.

Nor does it seem likely that the Memory Decoding system would behave in a fragile fashion if noise were introduced there. The way in which information is passed between the components of this system is already somewhat ‘noisy’: We have said that a simple Gaussian function is used to determine the way in which each cell in the Vector Decode subsystem will be inhibited or excited by the cells in the Memory Sum subsystem. Notice that a Gaussian function is used here because it is regarded by the authors as being a biologically plausible way for inhibitions and excitations to be arranged. (They cite a study of interneurons in the motor system of the leech in support of this.) Biological plausibility here comes at some small cost to accuracy since, from the point of view of path integration’s mathematics, a Gaussian function is not quite the tool for the job that this part of the system needs to accomplish. A distribution of inhibitory and excitatory synaptic weights that follows a *cosine* function would give a more accurate approximation of the path got by integrating the several vector fragments represented by the activity in the Memory Sum subsystem. The more biologically plausible Gaussian assignment of weights is a deviation from that ideal. It seems not to impair the performance of the system excessively. This gives some reason for thinking that here too the system would not be disproportionately disrupted by noise, since it is already tolerating some deviation from the ideal.

Gallistel and King’s final complaint is the most important one. It is also related to the idea that a connectionist memory system might be so fragile that the noisy realities of biological information processing would defeat it. This complaint is based on Gallistel and King’s idea that information can only be stored in an artificial neural network by passing it around a loop. In order for that method of information storage not to be highly susceptible to corruption of the information stored, it is necessary for the connections between the parts of that loop to preserve *all* of the information that is given to them (that is, the connections have to have a gain of one). Without a gain of one, the effect of being passed around the loop will be that the information stored in this memory system gets ever more corrupted with each pass around. But an uncorrupting gain of one is biologically quite implausible. Stochastic connections are, as we have said, the biological norm. Any biologically plausible system that preserves information by passing it around in a loop must therefore have some way to cope with the fact that that information will quickly become corrupted. If a system has no means to cope with that, and so essentially depends upon a loop with a gain of one, then it is not a biologically plausible model.

This last complaint might seem not to apply to the Bernardet et al. model at all, since that model preserves information in the pattern of activation in an array of persistently firing neurons, and so does not need to preserve information by passing it around in a loop. This use of persistent-firing as its mechanism for information storage might seem to be at the heart of the matter. We have seen that Gallistel and King take their criticism of the Samsonovich and McNaughton model to generalize to other connectionist models because they think that that model “relies on the *only* widely conjectured mechanism for performing the essential memory function, reverberatory loops.” (p. xv, emphasis added) And yet Bernardet et al. seem to be conjecturing some quite different mechanism for performing this essential memory function. They retain information in their network, not by passing it around in a reverberatory loop, but by employing cells that fire persistently. One might think that it is simply this that enables them to avoid the Gallistel and King objections. It is important to realize that that would not be quite correct, and that there is a genuine objection here needing to be dealt with.

Although the use of persistently firing neurons is of the first importance in the Bernardet et al. model, it would be misleading to regard such neurons as providing a clear alternative to reverberatory loops, and as having none of the problems that Gallistel and King show loops to bring with them. Their attempt to use an alternative to reverberatory loops does create a problem for Bernardet et al’s model, although it is not, ultimately, a problem that will prove to be insoluble.

The Memory subsystem of the Bernardet et al. model is composed of neurons that they describe as having “a high membrane persistence” (p. 166). It is the firing of these neurons that performs the function of memory. In support of the idea that persistently firing neurons are not biologically implausible, Bernardet et al. cite Major and Tank’s review article, which we quoted from above. The problem here is that the persistent firing that Major and Tank are concerned with has a duration that ranges “from hundreds of milliseconds to tens of seconds” (Major and Tank 2004, p. 675), whereas Gallistel and King speak of “a dead reckoning ant journeying over the desert floor for tens of minutes or a homing pigeon navigating by dead reckoning for tens of hours” (p. 246).

The problem that this disparity of durations creates is clear enough: If the Bernardet et al. system is able to model the path integration of a journey taking several minutes that is only because it is able to store information about the ant’s heading in the early parts of that journey for all of these several minutes. But the system’s way of storing that information is in persistently firing cells, and there is no biologically plausible mechanism by which cells could persist in firing for several minutes. The persistent firing mechanism of information storage therefore cannot be adequate to account for the ant’s behaviour. The Bernardet et al. model must therefore be adapted to incorporate some *other* mechanism that allows for information in the Memory system to be stored for longer durations. Over this longer timescale Gallistel and King are right that “the only widely conjectured mechanism for performing the essential memory function is reverberatory loops”. The information encoded in Bernardet et al’s memory subsystem, if it is be held for longer than a cell can persist in firing, must therefore be sent to some other system, which in turn passes it back. It must, therefore, be sent around a loop. This might seem to give the result that Bernardet et al. will need to change precisely that feature of their model that enabled it to avoid Gallistel and King’s last and most important objections. It might therefore seem that those objections have not been successfully avoided after all.

There is a genuine problem here, but in order to see how that problem might be solved, first notice why it is that a gain of less than one in a reverberatory circuit will lead to a rapidly corrupting memory. The problem here is that the information, having been corrupted by one trip around the reverberatory loop, will then be corrupted again by the next trip round, and so on. As Gallistel and King write:

if we actually built our machine out of real neurons, we would discover that we could not realize a neural loop with a noise-free gain of exactly 1, and that any departure from the value 1, no matter how small, was eventually disastrous. Suppose, for example, that the gain was 0.99 or 1.01 instead of 1.00. … Each time the circulating quantity goes around the loop it is diminished or augmented by the gain in the loop. With neural loops, the quantities circulate rapidly, let us say, 10 cycles per second. Thus at the end of 10 s, the circulating quantity is 0.99^100^ = 0.37 or 1.0001^100^ = 2.7, depending on whether the gain was slightly less or slightly more than 1. So our integrator is unrealistically parameter sensitive. (p. 258, ellipsis in original)

This problem makes it hopeless to attempt to store a real value by sending an analogue representation of that value around in a reverberatory loop. But, in adapting the Bernardet model so that it can cope with the integration of a journey that is longer than the duration of biologically plausible persistent firing, the information that we found ourselves needing to store in a reverberatory loop was not an analogue representation of a real value. The information that we needed to store was the pattern of persistent firings that have accumulated in the several columns of the Memory subsystem. The cells in that subsystem are like binary switches. They are either on or off, depending on whether their stochastic connection to the Gater subsystem has flipped them on or not. The information that needs to be preserved in a reverberatory loop is not an analogue representation of a real value. Its format is digital. Because of that a reverberatory loop *can* successfully maintain that information, in an uncorrupted form, without needing to be built from biologically implausible components with a gain of exactly one. A set of cells that fire whenever their input exceeds some threshold can preserve a pattern of digital information indefinitely, provided only that those cells are connected into a loop by connections having a gain that is sufficient to keep the strength of the transmitted signal above the firing threshold. Such cells and such connections are biologically plausible.

Gallistel and King’s last complaint is therefore avoided since there is now no threat of the reiterated corruption of information that creates the noise intolerance to which systems incorporating reverberatory loops are susceptible. Once we adjust Bernardet et al’s model to remove the implausible supposition of indefinitely persistent neuronal firing there will be a tendency for the oldest information in the Memory subsystem to leak out. Before too much of it does so, the pattern of firings in the memory subsystem needs to be sent off into a loop where it can be preserved for longer time scales. But, since the format of this information is digital, that loop can be implemented with biologically plausible neurons, having a noise-introducing gain. When a food source is found, and the direction back to the nest needs to be calculated, the Memory Sum subsystem can then add together whatever information is being held in these reverberatory loops, together with any information that is still represented in the form of persistent firing. The totals can then be sent to the Vector Decode system as before.

## The Significance of the Model

There are two features of the Bernardet et al. model that explain its ability to avoid the sorts of problems that Gallistel and King raise for Samsonovich and McNaughton. The first is that the Bernardet et al. model (like other more recent models of the same phenomenon, Cruse and Wehner 2011) avoids the use of large arrays to represent locations in the space through which the ant is navigating. These models operate without any explicit representation of locations in space at all (and so without any need to make any plausible or implausible assumption about the space’s topology). What they encode instead is a stochastically gathered sampling of the directions in which the ant has been progressing over the course of its journey. This needs relatively few neurons, and so avoids the issues of coordinating communication among large networks. It is also information that can be converted, by a biologically plausible mechanism, into a digital format, and that can therefore be preserved without loss, even in the presence of noise.

The incorporation of cells with a high ‘membrane persistence’ into the Bernardet et al. network has the effect of equipping that network with something like a memory stack. This is, to some extent, a vindication of Gallistel and King’s claim that the accomplishment of dead reckoning will require a read/write memory system, but it is a vindication that does not bear out the point that they ultimately want to argue for, which is that our current neuroscience does not have the resources to account for such a system, so that some kind of molecular memory system must still be awaiting discovery. Pattern coding in cells that fire persistently, but not indefinitely, when paired with the storage of that pattern in reverberatory loops, can avoid the problems that are associated with the sorts of systems that Gallistel and King consider, which attempt to make use of reverberatory loops alone.

The Bernardet et al. model is one of the simpler models of dead reckoning to have been proposed in the recent literature. Like all models at this level, it incorporates patent inaccuracies. It supposes, for example, that time passes in discrete steps, and that cells can be persistently turned on (albeit in a stochastic fashion) by a single excitatory input, coming from a single source. These inaccuracies are real, but they are not like the inaccuracies in Samsonovich and McNaughton’s model, of supposing that the space navigated by the ant has the topology of a torus, or that its brain devotes several billion neurons to the purposes of navigation. They are more like the legitimate elisions of detail that tractable modelling always requires. Such inaccuracies do not prevent the model from answering a ‘How possibly?’ question (Craver 2007), in a way that suggests the sort of discoveries that would need to be made if the elided details are to be filled in, so that a ‘How actually?’ answer can eventually be given. There is room for disagreement about whether the requisite discoveries are likely to be forthcoming, but Gallistel and King have not yet provided any principled reason to suppose that they could not be.

The success of the Bernardet et al. model shows that elements of the sort that are perfectly familiar to contemporary neuroscience – and that are therefore of the sort that the connectionist modeller can legitimately employ – are sufficient to account for a memory system that is good enough for the purposes of dead reckoning. This is only a very circumscribed victory for connectionism, but it is not without philosophical significance.

The early successes of connectionist modelling soon attracted the attention of philosophers (Dennett 1986; Clark 1989, 1993), some of whom had qualms about the connectionist approach (Fodor and Pylyshyn 1988; Fodor and McLaughlin 1990), and some of whom had qualms about those qualms (Chalmers 1993). This philosophical engagement with the connectionist programme tended to be concerned with the question of whether grounds could be found for the wholesale dismissal of the entire connectionist project (e.g., Chomsky, in Chomsky and Place 2000; Fodor 2001). In spite of those dismissals, there continues to be sophisticated research – including philosophically ambitious research – that employs connectionism’s methods (e.g., Suskever et al. 2011). Gallistel and King may very well be right that some of the ambitions of this research are misplaced, and that the brain’s more sophisticated psychological accomplishments must depend on an as yet undiscovered memory mechanism, different in kind from the sort of thing that is postulated by even the most sophisticated of connectionist models, but they have not established that such mechanisms are required for the explanation of dead reckoning. In order to understand how much can be built on connectionist foundations, it will continue to be necessary to engage in the normal scientific business of examining the merits and limitations of the connectionists’ particular explanatory proposals. Fully general grounds for a wholesale dismissal of connectionism have not yet been established.
